# Correlation of *UGT1A1* Gene Polymorphisms or Prior Irinotecan Treatment and Treatment Outcomes of Nanoliposomal-Irinotecan plus 5-Fluorouracil/Leucovorin for Pancreatic Ductal Adenocarcinoma: A Multicenter, Retrospective Cohort Study (HGCSG2101)

**DOI:** 10.3390/jcm12041596

**Published:** 2023-02-17

**Authors:** Kazuaki Harada, Takahiro Yamamura, Osamu Muto, Michio Nakamura, Susumu Sogabe, Kentaro Sawada, Shintaro Nakano, Masataka Yagisawa, Tetsuhito Muranaka, Masayoshi Dazai, Miki Tateyama, Yoshimitsu Kobayashi, Sosuke Kato, Kazuteru Hatanaka, Yasuyuki Kawamoto, Satoshi Yuki, Yuh Sakata, Naoya Sakamoto, Yoshito Komatsu

**Affiliations:** 1 Department of Gastroenterology and Hepatology, Hokkaido University Hospital, Sapporo 060-8638, Japan; 2 Department of Medical Oncology, Japanese Red Cross Akita Hospital, Akita 010-1495, Japan; 3 Department of Gastroenterology, Sapporo City General Hospital, Sapporo 060-8604, Japan; 4 Department of Medical Oncology, KKR Sapporo Medical Center, Sapporo 062-0931, Japan; 5 Department of Medical Oncology, Kushiro Rosai Hospital, Kushiro 085-8533, Japan; 6 Department of Gastroenterology, Iwamizawa Municipal General Hospital, Iwamizawa 068-8555, Japan; 7 Department of Medical Oncology, Japanese Red Cross Kitami Hospital, Kitami 090-8666, Japan; 8 Department of Internal Medicine, Wakkanai City Hospital, Wakkanai 097-8555, Japan; 9 Department of Gastroenterology, Sapporo Medical Center NTT EC, Sapporo 060-0061, Japan; 10 Department of Gastroenterology, Tomakomai Nissho Hospital, Tomakomai 053-0803, Japan; 11 Department of Gastroenterology, Hakodate Municipal Hospital, Hakodate 041-8680, Japan; 12 Division of Cancer Center, Hokkaido University Hospital, Sapporo 060-8638, Japan; 13 Department of Medical Oncology, Misawa City Hospital, Misawa 033-0022, Japan

**Keywords:** pancreatic cancer, nanoliposomal irinotecan, nal-IRI, UGT1A1, irinotecan

## Abstract

The effects of *UGT1A1* gene polymorphisms or prior irinotecan treatment on treatment outcomes of nanoliposomal-irinotecan plus 5-fluorouracil/leucovorin (nal-IRI+5-FU/LV) in patients with unresectable pancreatic ductal adenocarcinoma (PDAC) are not established. This multicenter, retrospective cohort study compared treatment outcomes in patients with UGT1A1*1/*1 and those with UGT1A1*1/*6 or *1/*28 genotypes. We also analyzed the impact of prior irinotecan treatment on survival outcomes in 54 patients treated with nal-IRI+5-FU/LV. Comparable effectiveness was found regardless of the *UGT1A1* genotypes. While no significant differences were found, grade ≥3 neutropenia and febrile neutropenia were more frequent in patients with UGT1A1*1/*6 or *1/*28 than in those with UGT1A1*1/*1 genotypes (grade ≥3 neutropenia, 50.0% vs. 30.8%, *p* = 0.24; febrile neutropenia, 9.1% vs. 0.0%, *p* = 0.20, respectively). No significant difference in progression-free survival (PFS) and overall survival (OS) was observed between irinotecan-naïve-patients and other patients. However, irinotecan-resistant patients showed significantly shorter PFS (hazard ratio (HR) 2.83, *p* = 0.017) and OS (HR 2.58, *p* = 0.033) than other patients. Our study indicated that patients with UGT1A1*1/*6 or *1/*28 may be prone to neutropenia, though further study is needed. The survival benefit of nal-IRI+5-FU/LV could be maintained in patients without disease progression after irinotecan therapy.

## 1. Introduction

Pancreatic cancer, of which the most common histological type is pancreatic ductal adenocarcinoma (PDAC), is the seventh-leading cause of cancer death worldwide [1]. The majority of PDAC cases present with unresectable (metastatic or locally advanced) disease upon diagnosis, with an overall 5-year survival rate of approximately 10% [2]. Curative treatment for unresectable PDAC is virtually impossible, and their prognosis is extremely poor. The genetic abnormalities or microenvironmental mechanisms involved in the development of PDAC are gradually being elucidated, and the development of molecular targeting therapies that directly target the relevant signaling and immune checkpoint molecules is expected [3]. At present, however, conventional cytotoxic anticancer drugs are still the mainstay of treatment for unresectable PDAC.

Nanoliposomal irinotecan (nal-IRI) consists of an irinotecan-free base encapsulated in liposome nanoparticles that maintain higher intra-tumoral levels of both irinotecan and its active metabolite SN-38 [4]. A global phase III trial (NAPOLI-1) showed that nal-IRI plus 5-FU/leucovorin (5-FU/LV) treatment significantly increased the median overall survival (OS) compared with the 5-FU/LV control arm for patients with metastatic PDAC refractory to gemcitabine-based therapy (6.1 and 4.2 months, respectively; an unstratified hazard ratio (HR) of 0.67; *p* = 0.012) **[5]**. The median progression-free survival (PFS) was also superior to that of the control arm (3.1 and 1.5 months, respectively; HR of 0.56; *p* = 0.0001). Based on these results, nal-IRI+5-FU/LV has been included in treatment guidelines as a recommended and approved option for use in patients with unresectable PDAC that progressed after gemcitabine-based chemotherapy [6,7,8].

Although nal-IRI+5-FU/LV is the recognized standard care for patients with pretreated PDAC, several clinical questions remain unanswered. First, the Japanese real-world data of patients with PDAC treated by nal-IRI+5-FU/LV are still scarce. As the Asian race is a significant predictor of neutropenia in patients receiving nal-IRI [9], it would be worthwhile to examine whether this therapy can be safely implemented in Japanese clinical practice.

Second, the impact of *UGT1A1* gene polymorphisms on the treatment outcomes of nalIRI+5-FU/LV is unclear. UGT1A1 is the liver enzyme that inactivates SN-38 to SN-38 glucuronide (SN-38G) [10]. More than 100 variants have been found in the *UGT1A1* gene and these genetic variants can affect enzymatic function, causing reduced metabolic capacity [11]. Thus, many studies have examined the relationship between *UGT1A1* polymorphisms and irinotecan-induced toxicity. Especially, the relationship between irinotecan toxicity and UGT1A1*28 (rs3064744) and UGT1A1*6 (rs4148323) has been well considered. Patients who are carriers of two decreased function alleles (UGT1A1*28/*28, *6/*6) experience delayed metabolism of SN-38 and achieve higher levels of SN-38 than those with UGT1A1*1/*1 who are predicted to be normal metabolizers of SN-38. Double heterozygous UGT1A1*6/*28 is also associated with delayed metabolism of SN-38 [11]. It has been shown that delayed metabolism of SN-38 leads to enhanced irinotecan-induced toxicity and patients with these *UGT1A1* genotypes are recognized as a higher risk population for irinotecan toxicity, such as neutropenia or diarrhea [10,11,12]. It is also known that a lower frequency of the UGT1A1*28 variant exists in Asian patients than in Caucasian patients. Meanwhile, the UGT1A1*6 variant is rare in Caucasian patients [10]. Thus, most of the national medicine authorities and guideline working groups in Western countries recommend a dose reduction of irinotecan for patients with UGT1A1*28/*28 [12,13,14,15]. In Japan, the Pharmaceuticals and Medical Devices Agency (PMDA) have recommended irinotecan dose reductions not only for patients with UGT1A1*28/*28, but also those with *6/*6 or *6/*28 [16]. It is recognized as necessary to confirm *UGT1A1* gene polymorphism before administration of irinotecan, and testing for UGT1A1*28 and *6 gene polymorphisms is available in Japanese clinical practice. On the other hand, it is controversial that the initial dose reduction of irinotecan is needed for patients who are heterozygous for one decreased functional allele (*1/*6 or *1/*28). Patients with UGT1A1 *1/*6 or *1/*28 are predicted to be intermediate metabolizer of SN-38 [11], and it is reported that they have intermediate risk for irinotecan-induced toxicity compared with patients with UGT1A1*1/*1 [17]. However, whether irinotecan-induced toxicity is increased in patients with UGT1A1*1/*6 or *1/*28 is inconclusive owing to various reports [11,18,19,20]. Moreover, the clinical significance of the difference between *1/*6 and *1/*28 is not fully understood.

Because most studies have focused on non-liposomal irinotecan formulations, the impact of *UGT1A1* polymorphisms on nal-IRI has been more unclear. In NAPOLI-1 trial, nal-IRI was administered at a reduced dose in patients with UGT1A1*28/*28 [5]. However, the impact of UGT1A1*1/*28 on treatment outcomes has not been fully investigated, and no information is available on UGT1A1*1/*6. In a Japanese phase 2 trial [5,21], dose reduction of nal-IRI was required for the patients with UGT1A1*28/*28, *6/*6, and *6/*28. On the other hand, patients with UGT1A1*1/*28 or *1/*6 received nal-IRI at the same dose as patients with UGT1A1*1/*1, and the impact of UGT1A1*1/*6 or *1/*28 on treatment outcome was not analyzed. If patients with UGT1A1*1/*6 or *1/*28 have the risk of toxicity of nal-IRI+5-FU/LV therapy, the management of this therapy may need to be reconsidered. In addition, the clinical data of patients with UGT1A1 double variants (*28/*28, *6/*6, *6/*28) are still scarce.

Finally, it is unclear whether nal-IRI+5-FU/LV therapy would be effective in patients with PDAC who have received irinotecan-containing chemotherapy, such as folinic acid, 5-fluorouracil, irinotecan, and oxaliplatin (FOLFIRINOX) [22]. Subgroup analysis of the NAPOLI-1 study revealed that nal-IRI did not have an additional survival benefit in patients who received irinotecan [23]. Moreover, OS was significantly shorter in patients who were refractory to irinotecan than in those with nonrefractory response [24]. FOLFIRINOX therapy is considered as one of the effective treatment options for patients with PDAC in Japan. However, its toxicity, including hematologic toxicity or sensory neuropathy, is often a reason to discontinue the treatment. Given that treatment options for PDAC are limited, it is worthy of consideration whether nal-IRI+5-FU/LV can be a treatment option for patients for in whom FOLFIRINOX therapy was discontinued for reasons other than disease progression.

In this multicenter, retrospective study, we evaluated real-world data of nal-IRI+5-FU/LV in Japanese patients with unresectable PDAC, including those previously treated with irinotecan-containing chemotherapy. We compared treatment outcomes in patients with UGT1A1*1/*1 and those with UGT1A1*1/*6 or *1/*28 genotypes. In addition, the impact of prior irinotecan treatment on the efficacy of nal-IRI+5-FU/LV was evaluated.

## 2. Materials and Methods

### 2.1. Patients and Study Design

This multicenter, retrospective, observational cohort study was conducted by the Hokkaido Gastrointestinal Cancer Study Group (HGCSG). We retrospectively reviewed the clinical data of patients with unresectable PDAC who initiated nal-IRI+5-FU/LV between June and December 2020 in any of the 10 participating institutions in Japan. Patients with histologically or cytologically confirmed PDAC were eligible for inclusion in this study if they had evidence of disease progression on prior chemotherapy, including neoadjuvant, adjuvant, or palliative chemotherapy. Patients with prior neoadjuvant or adjuvant therapy were counted as having one prior line of chemotherapy if their disease had progressed within 6 months of the end of the prior neoadjuvant or adjuvant therapy. The electronic medical history was queried for patient demographics, Eastern Cooperative Oncology Group performance status (ECOG PS), *UGT1A1* status, carbohydrate antigen 19-9 level at baseline, details of treatments before nal-IRI+5-FU/LV, starting date of nal-IRI+5-FU/LV, treatment starting dose, treatment dose reductions, treatment duration, adverse events, PFS, and OS. PFS was defined as the time from initiation of nal-IRI+5-FU/LV treatment until objective tumor progression or death, whichever occurs first. OS was defined as the time from the start of first nal-IRI+5-FU/LV administration to death. Generally, radiological tumor evaluation was performed by computed tomography every 6–12 weeks after treatment initiation by physicians’ judgement. Tumor response was evaluated using the Response Evaluation Criteria In Solid Tumors version 1.1. Patients who presented obvious clinical disease progression were classified as having progressive disease. Adverse events were graded using the Common Toxicity Criteria for Adverse Events ver. 5.0. The relative dose intensity (RDI) was defined as the average dose, adjusting for body surface area during the entire treatment course.

### 2.2. UGT1A1 Testing

Pharmacogenetic analysis by *UGT1A1* testing was recommended as routine clinical practice for all patients receiving nal-IRI treatment. UGT1A1*28 and UGT1A1*6 gene polymorphisms were analyzed using Invader technology. The absence of both UGT1A1*6 and UGT1A1*28 was defined as UGT1A1*1/*1.

### 2.3. Nal-IRI + 5-FU/LV Treatment

The nal-IRI+5-FU/LV regimen consisted of 70 mg/m^2^ nal-IRI (equivalent to 80 mg/m^2^ of irinotecan salt base) administered by intravenous infusion over 90 min. This was followed by 200 mg/m^2^ *l*-LV via intravenous infusion over 2 h and then 2400 mg/m^2^ 5-FU via intravenous infusion over 46 h every 2 weeks. Chemotherapy dose and schedule adjustments were allowed with the investigator’s judgment. The treatment was continued until disease progression, occurrence of unacceptable toxicity, or patient’s refusal to continue.

### 2.4. Statistical Analysis

Data were presented with 95% confidence intervals calculated using standard methods based on a binomial distribution. Survival analyses were performed with the Kaplan–Meier method. A log-rank test and a Cox proportional hazard model were used to compare patients according to subgroups. A multivariate Cox proportional hazards model was also used to identify the effects of clinical factors on PFS and OS. We tested the proportional hazards assumption by EZR ver1.61, which is for R. More precisely, it is a modified version of R commander designed to add statistical functions frequently used in biostatistics [25]. The frequency of adverse events was compared using Fisher’s exact test between patients with wild-type *UGT1A1* (wild-type group) and patients with single-heterozygous *UGT1A1* (single-heterozygous group). All analyses except the test for the proportional hazards assumption were performed using JMP ver14 software (SAS Institute Inc., Care, NC, USA).

### 2.5. Ethics

The study design and protocol were approved by the institutional review board of Hokkaido University Hospital and all other participating institutions. The need for informed consent was waived owing to the retrospective nature of the study. This study was announced on a website (https://www.huhp.hokudai.ac.jp/date/rinsho-johokokai/approval/2021-7/, accessed on 13 February 2023).

## 3. Results

### 3.1. Patient Characteristics

A total of 54 patients with unresectable PDAC who received at least one dose of nal-IRI+5-FU/LV were included. The median follow-up time, from the date of starting treatment to the date of cutoff on 31 June 2021, was 9.7 months. All patients were histologically or cytologically diagnosed with PDAC. The baseline clinicopathological characteristics are listed in Table 1. Their median age was 68 (range 46–77) years and 30 patients were male (55.6%). Nearly all patients (*n* = 50, 92.6%) presented with metastatic disease at the start of treatment with nal-IRI plus 5-FU/LV. The most common metastatic site was the liver (*n* = 33, 61.1%), followed by the lymph nodes (*n* = 26, 48.1%) and peritoneum (*n* = 15, 27.8%). Eleven patients (20.4%) had ≥3 metastatic sites. Nearly all patients had an ECOG PS of 0 (*n* = 26, 48.1%) or 1 (*n* = 25, 46.3%), whereas 3 (5.6%) patients had an ECOG performance status of 2. Moreover, 32 (59.3%) patients had received one previous line of chemotherapy, and 10 (18.5%) were treated with ≥3 lines of chemotherapy. Furthermore, 13 (24.0%) patients had been treated with irinotecan-containing chemotherapy before nal-IRI+5-FU/LV administration. Among them, seven patients discontinued FOLFIRINOX, mainly because of adverse events, such as neutropenia or peripheral sensory neuropathy, without disease progression. The details of administered chemotherapy regimens before nal-IRI+5-FU/LV are listed in Table S1. *UGT1A1* genotype testing was performed in 51 (94.4%) patients. Among them, 26 (48.1%) had UGT1A1*1/*1, 22 (40.7%) had heterozygous *UGT1A1*(*1/*28 or *1/6*), and 3 (5.6%) had double variants (UGT1A1*6/*28 or 6*/6*).

### 3.2. Treatment Outcomes for All Patients

The median number of treatment cycles was 5 (range, 1–22). At data cutoff, 8 (15.0%) patients were undergoing nal-IRI+5-FU/LV treatment. Thirty-four patients (63.0%) started with the full recommended dose of 70 mg/m^2^ nal-IRI, whereas 20 (37.0%) patients started with the reduced dose. After starting nal-IRI+5-FU/LV treatment, 20 (37.0%) patients had a reduced nal-IRI dose, mainly because of neutropenia (22.2%), anorexia (13.0%), and fatigue (5.6%). Nine patients (16.7%) had reduced initial doses of 5-FU. Fourteen patients (25.9%) needed a 5-FU dose reduction mainly because of neutropenia (11.1%) and anorexia (11.1%). The median RDI of nal-IRI and 5-FU was 0.74 (range, 0.35–0.99) and 0.81 (range, 0.45–1.00), respectively. Treatment details for all patients are listed in Table S2.

**Figure 1:**
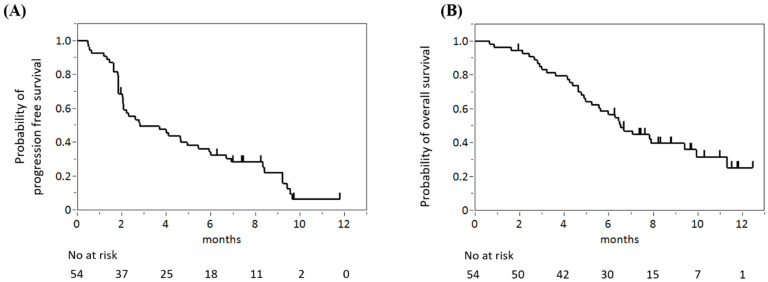
Progression-free survival (**A**) and overall survival (**B**) of all analyzed patients.

The median PFS was 2.8 months (95% CI 2.0–5.4) and the median OS was 6.6 months (95% CI 5.0–9.4) (Figure 1A,B). The response evaluation for patients with evaluable target lesions (*n* = 48, 89%) showed a partial response in 6 patients, stable disease in 20, and progressive disease in 21. Response and disease control rates were 12.5% (95% CI 3.1–25.9) and 54.2% (95% CI 40.0–68.3), respectively (Table 2).

The adverse events observed during nal-IRI+5-FU/LV treatment are listed in Table 3. Any-grade adverse events were observed in almost all patients (*n* = 53, 98%) and grade 3–4 adverse events were observed in 26 (48%) patients. The most common adverse events were lymphocytopenia (*n* = 52, 96%), anemia (*n* = 52, 96%), and fatigue (*n* = 40, 72%). The most frequent severe adverse events (grade ≥ 3) in our cohort were neutropenia (*n* = 22, 41%), leucopenia (*n* = 13, 24%), and lymphocytopenia (*n* = 13, 34%). Only one patient discontinued nal-IRI+5-FU/LV because of adverse events (interstitial pneumonia). No treatment-related adverse events resulted in death.

### 3.3. Comparison of Treatment Outcomes between UGT1A1*1/*1 and UGT1A1*1/*6 or *1/*28

We compared the treatment outcomes of nal-IRI+5-FU/LV in the UGT1A1*1 group (*n* = 26) and heterozygous group (*1/*28, *1/*6, *n* = 22). Treatment details by *UGT1A1* status are shown in Table 4. More patients in the heterozygous group started nal-IRI at a reduced dose (*n* = 12, 54.5%) than patients in the wild-type group (*n* = 6, 23.1%). Moreover, the heterozygous group tended to reduce their nal-IRI dose more frequently, despite the lower starting dose of nal-IRI in subsequent treatment cycles. In addition, 10 (45.5%) patients in the single-heterozygous group and 8 (30.8%) in the wild-type group had a dose reduction of nal-IRI. However, these differences were not statistically significant (starting dose reduction, *p* = 0.13; dose reduction in subsequent cycles, *p* = 0.23). The median RDI values of nal-IRI were 0.79 (range, 0.43–0.99) in the wild-type group and 0.69 (range, 0.55–0.96) in the single-heterozygous group (*p* = 0.16).

Although no significant differences were found in adverse events, grade ≥3 neutropenia and febrile neutropenia were more frequent in the single-heterozygous group (Table 5). The rate of grade ≥3 neutropenia was 30.8% in the UGT1A1*1/*1 group and 50.0% in the heterozygous group (*p* = 0.24). Febrile neutropenia was observed in 9.1% of patients in the heterozygous group, but not in the UGT1A1*1/*1 group (*p* = 0.20). No significant differences were found between patients with UGT1A1*1/*28 and those with *1/*6 in the frequency of grade ≥3 neutropenia (50.0% vs. 50.0%, *p* = 1.00) and febrile neutropenia (10.0% and 8.3%, *p* = 1.00).

**Figure 2:**
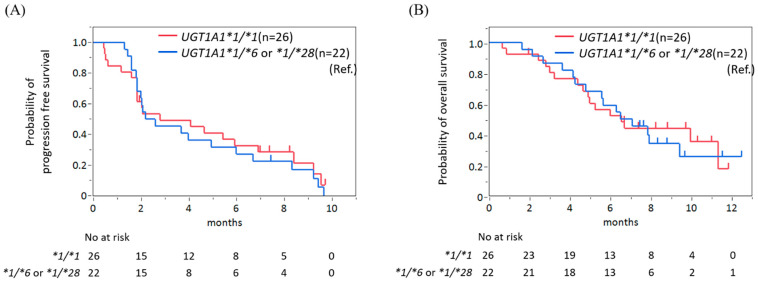
No significant difference in progression-free survival (**A**) and overall survival (**B**) was found between patients with wild-type *UGT1A1* and those with single-heterozygous *UGT1A1*.

No significant differences were noted in PFS and OS between the UGT1A1 *1/*1 group and heterozygous group (Figure 2A,B). The median PFS periods were 2.8 months (95% CI 1.9–6.9) in the UGT1A1*1/*1 group and 2.4 months (95% CI 1.9–6.0) in the heterozygous group (HR 0.86, 95% CI 0.47–1.59, *p* = 0.63). The median OS periods were 6.6 months (95% CI 4.7–11.3) in the UGT1A1*1/*1 group and 6.8 months (95% CI 4.3–9.4) in the heterozygous group (HR 0.96, 95% CI 0.46–1.96, *p* = 0.90).

To correct for potential confounding factors that have an impact on survival outcomes, we have also performed multivariate analysis with a Cox proportional hazard model. Clinical factors associated with survival in patients treated with nal-IRI+5-FU/LV (i.e., performance status, age, CA19-9 levels, neutrophil-to-lymphocyte ratio (NLR), and liver metastases) [26] were considered as explanatory variables. In this analysis, patients with good PS (ECOG PS0) had significantly better PFS (HR 0.39, 95% CI 0.19–0.80, *p* = 0.01) and OS (HR 0.37, 95% CI 0.15–0.90, *p* = 0.03) compared with other patients. Conversely, patients with high NLR (NLR > 5) had worse PFS (HR 2.67, 95% CI 1.04–6.88, *p* = 0.014) and OS (HR 4.55, 95% CI 1.38–14.95, *p* = 0.01) compared with other patients (NLR ≤ 5). However, it has been shown that UGT1A1 status had no statistically significant impact on either PFS (HR 1.14, 95% CI 0.59–2.22, *p* = 0.69) or OS (HR 0.88, 95% CI 0.41–1.90, *p* = 0.75) (Table 6).

There were no significant differences in PFS and OS between patients with UGT1A1*1/*6 and those with UGT1A1*1/*28 (Figure S1A,B). The median PFS periods were 4.3 months (95% CI 1.3–9.2) in patients with UGT1A1*1/*28 and 2.1 months (95% CI 1.6–6.0) in those with UGT1A1*1/*6 (*p* = 0.31). The median OS periods were 8.2 months (95% CI 1.6–N.R.) in patients with UGT1A1*1/*28 and 6.4 months (95% CI 2.6–7.9) in those with UGT1A1*1/*6 (*p* = 0.43), respectively.

### 3.4. Treatment Outcomes for Patients with UGT1A1 Double Variants

All three patients with *UGT1A1* double variants (*28/*28, *6/*6, *6/*28) were initiated nal-IRI with reduced doses (42–50 mg/m^2^). The median RDI of nal-IRI was 0.53 (range, 0.35–0.58) (Table S3). The frequency of grade ≥3 neutropenia and febrile neutropenia in patients with *UGT1A1* double variants was high (66.7% and 33.3%, respectively) (Table S4). The median PFS (95% CI 2.3–N.R.) and OS (95% CI 2.9–N.R.) were not reached (Figure S2A,B).

### 3.5. Correlation of Efficacy and Prior Irinotecan Exposure

**Figure 3:**
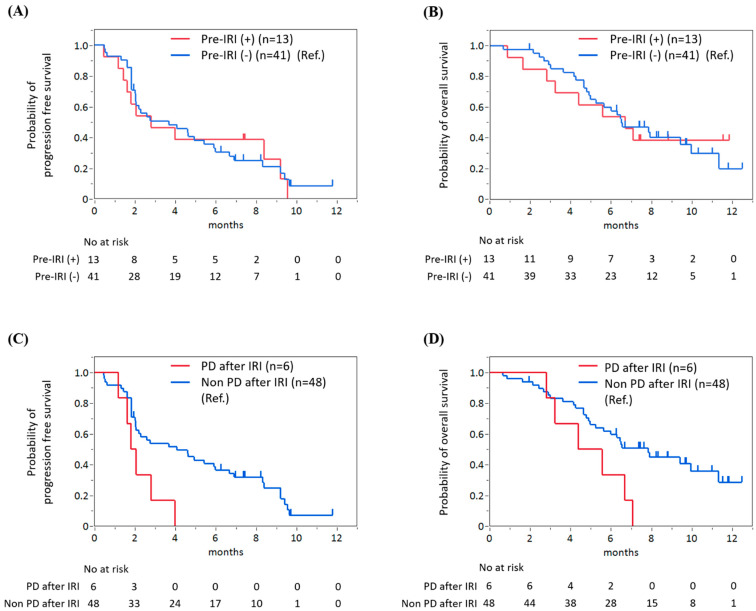
No significant difference in progression-free survival (PFS) (**A**) and overall survival (OS) (**B**) was found between patients with and without prior irinotecan therapy. However, patients with disease progression after irinotecan-containing chemotherapy had significantly shorter PFS (**C**) and OS (**D**) than other patients.

When comparing patients who previously received irinotecan (Pre-IRI(+), *n* = 13) to irinotecan-naïve patients (Pre-IRI(−), *n* = 41), no significant differences in PFS and OS were found between the two groups (Figure 3A,B). The median PFS periods were 2.8 months (95% CI 0.2–5.4) in Pre-IRI(+) patients and 3.7 months (95% CI 0.9–6.5) in Pre-IRI(−) patients (HR 1.1, 95% CI 0.5–2.1, *p* = 0.86). The median OS periods were 6.7 months (95% CI 2.8–N.R.) in Pre-IRI(+) patients and 6.5 months (95% CI 5.0–10.0) in Pre-IRI(−) patients (HR 1.01, 95% CI 0.43–2.15, *p* = 0.97), respectively. However, patients with disease progression after irinotecan-containing chemotherapy had shorter PFS and OS than other (Pre-IRI(−) and Pre-IRI(+) without disease progression) patients (Figure 2C,D). The median PFS periods were 1.9 months (95% CI 1.2–4.0) in patients with disease progression after irinotecan-containing chemotherapy and 4.1 months (95% CI 2.0–6.0) in other patients (HR 2.83, 95% CI 1.04–6.56, *p* = 0.017), respectively. The median OS periods were 5.0 months (95% CI 2.8–7.1) in patients with disease progression after irinotecan-containing chemotherapy and 7.8 months (95% CI 5.2–11.3) in other patients (HR 2.58, 95% CI 0.95–5.98, *p* = 0.033). A multivariate analysis showed patients with PD after irinotecan had significantly worse PFS compared with patients with non-PD after irinotecan (HR 2.84, 95% CI 1.10–7.37, *p* = 0.03). Although not statistically significant, OS tended to be worse in patients with PD after irinotecan compared with other patients (HR 2.50, 95% CI 0.96–6.47, *p* = 0.06). Patients with high NLR (NLR > 5) had worse PFS (HR 2.60, 95% CI 1.06–6.40, *p* = 0.04) and OS (HR 3.38, 95% CI 1.22–9.32, *p* = 0.02) compared with other patients (NLR ≤ 5). Good performance status (ECOG PS 0) was significantly related to better PFS (HR 0.43, 95%CI, 0.23–0.83, *p* = 0.01), but not significantly associated with OS (HR 0.51, 95% CI 0.24–1.12, *p* = 0.09) (Table 7).

## 4. Discussion

Compared with previous pivotal clinical trials [5,21], our patients tend to be older and heavily pretreated, with some patients receiving three or more lines of chemotherapy before treatment with nal-IRI+5-FU/LV (18.5%). Despite the clinical fragility in this real-world patient population, nal-IRI+5-FU/LV had a similar effectiveness in terms of OS and PFS, as reported in NAPOLI-1 [5] and Japanese phase 2 trials [21]. The safety profile is almost comparable to previous data in Asian patients [21,27,28]. Our data confirmed that nal-IRI+5-FU/LV for Japanese patients with PDAC is effective and well-tolerated in clinical practice.

The effect of *UGT1A1* on the toxicity of nal-IRI, especially UGT1A1 *1/*28 or *1/*6, has not been fully understood. To date, few studies and guidelines have mentioned the effect of UGT1A1 *1/*28 or *1/*6 genotype on the risk of irinotecan-induced toxicity. However, a meta-analysis by Yang et al. indicated that patients with these polymorphisms have an intermediate risk for severe neutropenia compared with patients with UGT1A1*1/*1 who have normal ability to metabolize irinotecan [17]. Despite the even fewer reports on the association between nal-IRI toxicity and single-heterozygous *UGT1A1*, Roy et. al. reported that grade ≥3 neutropenia occurred more frequently in patients who were heterozygous for UGT1A1*6 allele, while not for *28 allele in gastric cancer patients treated with nal-IRI [29]. Based on these reports, patients with heterozygous *UGT1A1*, especially *1/*6, appear to be at increased risk of nal-IRI-induced neutropenia. Racial differences in the frequency of the UGT1A1*6 variant have been reported, with a higher frequency in Asians than in Caucasians [30]. Previous clinical studies have revealed that Asians had more neutropenia but less diarrhea than Caucasians [9,31]. Although no studies have directly demonstrated this, the higher frequency of neutropenia in Asians may be influenced by racial differences in *UGT1A1*. In our analysis, the heterozygous group had a higher incidence of severe neutropenia and febrile neutropenia than patients with UGT1A1 *1/*1, requiring a further reduction in nal-IRI, even though many of them had received a reduced starting dose of nal-IRI. These findings may support the finding that patients with UGT1A1 *1/*6 or *1/*28 are at higher risk of nal-IRI-induced severe neutropenia than those with UGT1A1*1/*1. If UGT1A1*1/*6 and *1/*28 are the risk factor of neutropenia induced by nal-IRI, reconsidering the administration dose of nal-IRI by *UGT1A1* status will be necessary. In addition, considering *UGT1A1* heterozygosity as a risk factor for neutropenia may allow for a more accurate evaluation of the safety of nal-IRI-containing chemotherapy in future clinical trials. This may help to determine the appropriate nal-IRI dose for each patient. However, our study revealed no significant differences in the frequency of neutropenia between the UGT1A1*1/*1 and heterozygous groups, and caution should be exercised when interpreting the results. As our study was a small cohort retrospective study and was not adjusted for other factors (e.g., starting dose of nal-IRI), further investigation is needed to determine the effect of UGT1A1*1/*28 or *1/*6 on neutropenia related to nal-IRI+5-FU/LV.

Though many of the patients with UGT1A1*1/*28 or *1/*6 had received a reduced starting dose of nal-IRI, their PFS and OS were comparable to those in patients with UGT1A1*1/*1. Several reports from Asian countries have suggested that the initial dose reduction of nal-IRI results in less frequent neutropenia but no change in efficacy [32,33]. It is possible that the high number of Asian patients with the UGT1A1*6 variant may result in adequate drug exposure even with reduced doses of nal-IRI. The initial dose reduction of nal-IRI may be a treatment option, especially in Asians who appear to be more prone to nal-IRI-related neutropenia.

Few studies have reported clinical data on nal-IRI+5-FU/LV in patients with *UGT1A1* double variants. Our study included two patients with UGT1A1*6/*6 and one patient with UGT1A1*6/*28. As in the Japanese Phase II study [21], these patients had a high frequency of grade ≥3 neutropenia and febrile neutropenia, even though all three patients had started with a reduced dose (42–50 mg/m^2^) of nal-IRI. Although our results are based on a small number of patients, we believe that patients with *UGT1A1* double variants require further attention to neutropenia, even with a reduced dose of initial nal-IRI. Although patients with *UGT1A1* double variants required an intensive dose reduction of nal-IRI, they were able to continue nal-IRI+5-FU/LV. Therefore, it is not necessary to avoid nal-IRI in general because of *UGT1A1* double variants.

In this study, patients with disease progression after irinotecan-containing chemotherapy had significantly shorter PFS and OS than other patients. On the contrary, no significant difference in PFS and OS was found between patients with and without prior irinotecan therapy. These results indicate that nal-IRI+5-FU/LV is also effective in patients receiving irinotecan if there is no disease progression after irinotecan therapy, as already reported by Smith et al. [24]. While FOLFIRINOX is effective for PDAC, it has been suggested to be more toxic than nal-IRI+5-FU/LV [27,28]. Given the tolerable safety profile of nal-IRI+5-FU/LV, this may be a promising treatment option for patients who cannot continue FOLFIRINOX because of toxicity such as peripheral sensory neuropathy.

This study has several limitations that are also common in other real-world data analyses. First, all data were retrospectively extracted from medical records, which may not be as comprehensive and accurate as those from prospective clinical trials. Dose modification and radiological tumor evaluation intervals were left to the discretion of the physicians, not according to any specified protocol. This could affect the results of the efficacy analysis and result in a potential selection or recall bias. Second, the relatively small sample size limits the interpretation of the subgroup analysis such as *UGT1A1* status. To confirm the impact of *UGT1A1* status or previous irinotecan exposure on the efficacy and safety profile of nalIRI+5-FU/LV treatment, we consider that a comparative prospective study with a larger population is needed. In that future study, treatment outcomes should be compared according to a specified protocol that defines the radiological tumor evaluation intervals, starting dose, and dose reduction criteria for nal-IRI. In addition, genetic alterations and microenvironments associated with progression of PDAC are now gradually becoming clear [3]. Future clinical trials will need to more comprehensively capture the mechanisms of pancreatic cancer progression and consider more effective therapeutic strategies.

## 5. Conclusions

Our study confirmed the clinical benefit of nal-IRI+5-FU/LV for Japanese patients with PDAC in a real-world setting. It is suggested that not only patients with UGT1A1 double variants but also patients with UGT1A1*1/*28 or *1/*6 may be prone to neutropenia. Further study is needed to determine the effect of *UGT1A1* genotype on the treatment outcomes of nal-IRI+5-FU/LV for patients with PDAC. Nal-IRI+5-FU/LV may be effective even if irinotecan has been administered in the past, as long as disease progression is not observed during irinotecan therapy.

## Figures and Tables

**Table 1:** Patient characteristics.

*n* = 54		*n*	(%)
Gender	Male Female	30 24	(55.6) (44.4)
Age	Median (range)	68 (46–77)	
ECOG PS	0 1 2	26 25 3	(48.1) (46.3) (5.6)
Primary tumor site	Head Body—Tail	28 26	(51.9) (48.1)
Disease status	UR-LA * UR-M ** Recurrence	4 33 17	(7.4) (61.1) (31.5)
Prior irinotecan-containing Cx ***	Yes No	13 41	(24.1) (75.9)
Prior lines of Cx ***	1 2 ≥3	32 12 10	(59.3) (22.2) (18.5)
Liver metastasis	Yes No	33 21	(61.1) (38.9)
Lymph node metastasis	Yes No	26 28	(48.1) (51.9)
Peritoneum metastasis	Yes No	15 39	(27.8) (72.2)
Lung metastasis	Yes No	14 40	(25.9) (74.1)
The number of metastasis sites	≤2 ≥3	43 11	(79.6) (20.4)
*UGT1A1* genotype	*1/*1 *1/*6 *1/*28 *6/*6 *6/*28 Unknown	26 12 10 2 1 3	(48.1) (22.2) (18.5) (3.7) (1.9) (5.7)
CA19-9	Median (range)	1742.7 (0–269,847.9)	

* unresectable-locally advanced. ** unresectable-metastatic. ***chemotherapy.

**Table 2:** Tumor response in patients with evaluable target lesions.

	*n* = 48
**Best response**	
Complete response	0
Partial response	6
Stable disease	20
Progressive disease	21
Not evaluated	1
Response rate [95% CI]	12.5% [3.1–21.9]
Disease control rate [95% CI]	54.2% [40.0–68.3]

**Table 3:** Adverse events for all patients.

*n* = 54	All Grade *n* (%)	≥Grade 3 *n* (%)
**Hematological adverse events**		
Leucopenia	30 (55.6)	13 (24.1)
Neutropenia	34 (63.0)	22 (40.7)
Anemia	52 (96.2)	7 (13.0)
Thrombocytopenia	19 (35.2)	2 (3.7)
**Non-hematological adverse events**		
AST increased	19 (35.2)	3 (5.6)
ALT increased	24 (44.4)	0
Blood bilirubin increased	4 (7.4)	0
Creatinine increased	11 (20.4)	1 (1.9)
Fatigue	40 (74.1)	3 (5.6)
Nausea	35 (64.8)	4 (7.4)
Vomiting	8 (14.8)	1 (1.9)
Anorexia	36 (42.6)	5 (9.3)
Diarrhea	25 (66.7)	1 (1.9)
Febrile neutropenia	3 (5.6)	3 (5.6)
Alopecia	23 (42.6)	0
Mucositis oral	7 (13.0)	0
Intestinal lung disease	1 (1.9)	0
Biliary tract infection	2 (3.7)	2 (3.7)

**Table 4:** Treatment details for nal-IRI according to *UGT1A1* status.

*n* (%)		*1/*1 (*n* = 26)	*1/*28 or *1/*6 (*n* = 22)	*p*-Value
Starting dose	Full Reduced	20 (76.9) 6 (23.1)	10 (45.5) 12 (54.5)	0.13
Starting dose (range, mg/m^2^)	Median Mean	70.0 (42–70) 65.8 (42–70)	70.0 (31–70) 63.2 (31–70)	0.28
Dose reduction in subsequent cycles	1 ≥2	7 (27.0) 1 (3.8)	10 (45.5) 0	0.23 1.00
Reason for dose reduction	Neutropenia Anorexia Fatigue Other	4 (15.4) 3 (11.5) 2 (7.7) 1 (3.8)	5 (22.7) 3 (13.6) 0 4 (18.2)	
Relative dose intensity	Median (range)	0.79 (0.43–0.99)	0.69 (0.55–0.96)	0.16

**Table 5:** Adverse events by UGT1A1 status.

*n* (%)	*1/*1 (*n* = 26)		*1/*28 or *1/*6 (*n* = 22)		*p*-Value	
	All Grade	≥Grade3	All Grade	≥Grade3	All Grade	≥Grade3
Hematological adverse events						
Leucopenia	14 (53.8)	5 (19.2)	13 (59.1)	6 (27.3)	0.78	0.73
Neutropenia	16 (61.5)	8 (30.8)	14 (63.6)	11 (50.0)	1.00	0.24
Anemia	25 (96.2)	4 (15.4)	21 (95.5)	3 (13.6)	1.00	1.00
Thrombocytopenia	10 (38.5)	1 (3.8)	6 (27.3)	1 (4.5)	0.54	1.00
Non-hematological adverse events						
AST increased	8 (30.8)	0	9 (40.9)	3 (13.6)	0.55	0.09
ALT increased	10 (38.5)	0	13 (59.1)	0	0.25	-
Blood bilirubin increased	2 (7.7)	0	2 (9.1)	0	1,00	-
Creatinine increased	5 (19.2)	1 (3.8)	5 (22.7)	0	1.00	1.00
Fatigue	19 (73.1)	2 (7.7)	15 (68.2)	1 (4.5)	0.76	1.00
Nausea	18 (69.2)	2 (7.7)	12 (54.5)	2 (9.1)	0.37	1.00
Vomiting	2 (7.7)	0	5 (22.7)	1 (4.5)	0.22	0.46
Anorexia	17 (65.4)	2 (7.7)	14 (63.6)	3 (13.6)	1.00	0.65
Diarrhea	13 (50.0)	0	9 (40.9)	0	0.57	-
Febrile neutropenia	-	0	-	2 (9.1)	-	0.20
Alopecia	12 (46.2)	-	8 (36.4)	-	0.57	-
Mucositis oral	4 (15.4)	0	3 (13.6)	0	1.00	-
Intestinal lung disease	1 (3.8)	0	0	0	1.00	-
Biliary tract infection	1 (3.8)	1 (3.8)	1 (4.5)	1 (4.5)	1.00	1.00

**Table 6:** Multivariate analysis for PFS and OS in *UGT1A1* analysis set (*n* = 48).

*n* = 48	Progression-Free Survival			Overall Survival		
	HR	95% CI	*p*-Value	HR	95% CI	*p*-Value
UGT1A1 status [*1/*1 vs. *1/*6 or *1/*28]	1.14	0.59–2.22	0.69	0.88	0.41–1.90	0.75
ECOG PS [0 vs. 1–2 (ref)]	0.39	0.19–0.80	0.01	0.37	0.15–0.90	0.03
Age [>65 vs. ≤65 (ref)]	1.15	0.61–2.20	0.65	1.67	0.72–3.82	0.24
Liver metastasis [Yes vs. No (ref)]	1.61	0.82-–3.15	0.16	1.63	0.74–3.61	0.23
NLR [>5 vs. ≤5(ref)]	2.67	1.04–6.88	0.04	4.55	1.38–14.95	0.01
Baseline CA19-9 levels [≥59*ULN vs. <59*ULN (ref)]	1.58	0.79–3.15	0.19	1.46	0.68–3.15	0.33
				ULN: upper limit of normal		

**Table 7:** Multivariate analysis for PFS and OS in prior-irinotecan analysis set (*n* = 54).

*n* = 54	Progression-Free Survival			Overall Survival		
	HR	95% CI	*p*-Value	HR	95% CI	*p*-Value
Disease progression after irinotecan treatment [PD after IRI vs. Non-PD after IRI(ref)]	2.84	1.10–7.37	0.03	2.50	0.96–6.47	0.06
ECOG PS [0 vs. 1–2 (ref)]	0.43	0.23–0.83	0.01	0.51	0.24–1.12	0.09
Age [>65 vs. ≤65 (ref)]	0.83	0.45–1.55	0.57	1.50	0.70–3.25	0.30
Liver metastasis [Yes vs. No (ref)]	1.41	0.72–2.77	0.31	1.53	0.69–3.40	0.29
NLR [>5 vs. ≤5 (ref)]	2.60	1.06–6.40	0.04	3.38	1.22–9.32	0.02
Baseline CA19-9 levels [≥59*ULN vs. <59*ULN (ref)]	1.69	0.88–3.26	0.12	1.54	0.74–3.22	0.25
				ULN: upper limit of normal		

## Data Availability

The data presented in this study are available upon reasonable request from the corresponding author. The data are not publicly available for privacy reasons.

## Supplementary Materials

The following supporting information can be downloaded at https://www.mdpi.com/article/10.3390/jcm12041596/s1. Table S1. Chemotherapy before nal-IRI+5-FU/LV, Table S2. Treatment details for all patients, Table S3. Treatment details for patients with UGT1A1 double variants, Table S4. Adverse events for patients with UGT1A1 double variants, Figure S1. Progression-free survival (A) and overall survival (B) in patients with UGT1A1*1/*6 and in those with UGT1A1*1/*28, Figure S2. Progression-free survival (A) and overall survival (B) of patients with UGT1A1 double variants.
