# Supplementary material for: Correlation of UGT1A1 Gene Polymorphisms or Prior Irinotecan Treatment and Treatment Outcomes of Nanoliposomal-Irinotecan plus 5-Fluorouracil/Leucovorin for Pancreatic Ductal Adenocarcinoma: A Multicenter, Retrospective Cohort Study (HGCSG2101)
Source: J Clin Med. 2023 Feb 17;12(4):1596. doi: 10.3390/jcm12041596 (PMC9963652; doi:10.3390/jcm12041596)
Supplement: Supplementary file 1 [file jcm-12-01596-s001.zip › jcm-2187982-supplementary.pptx]

## Slide 1
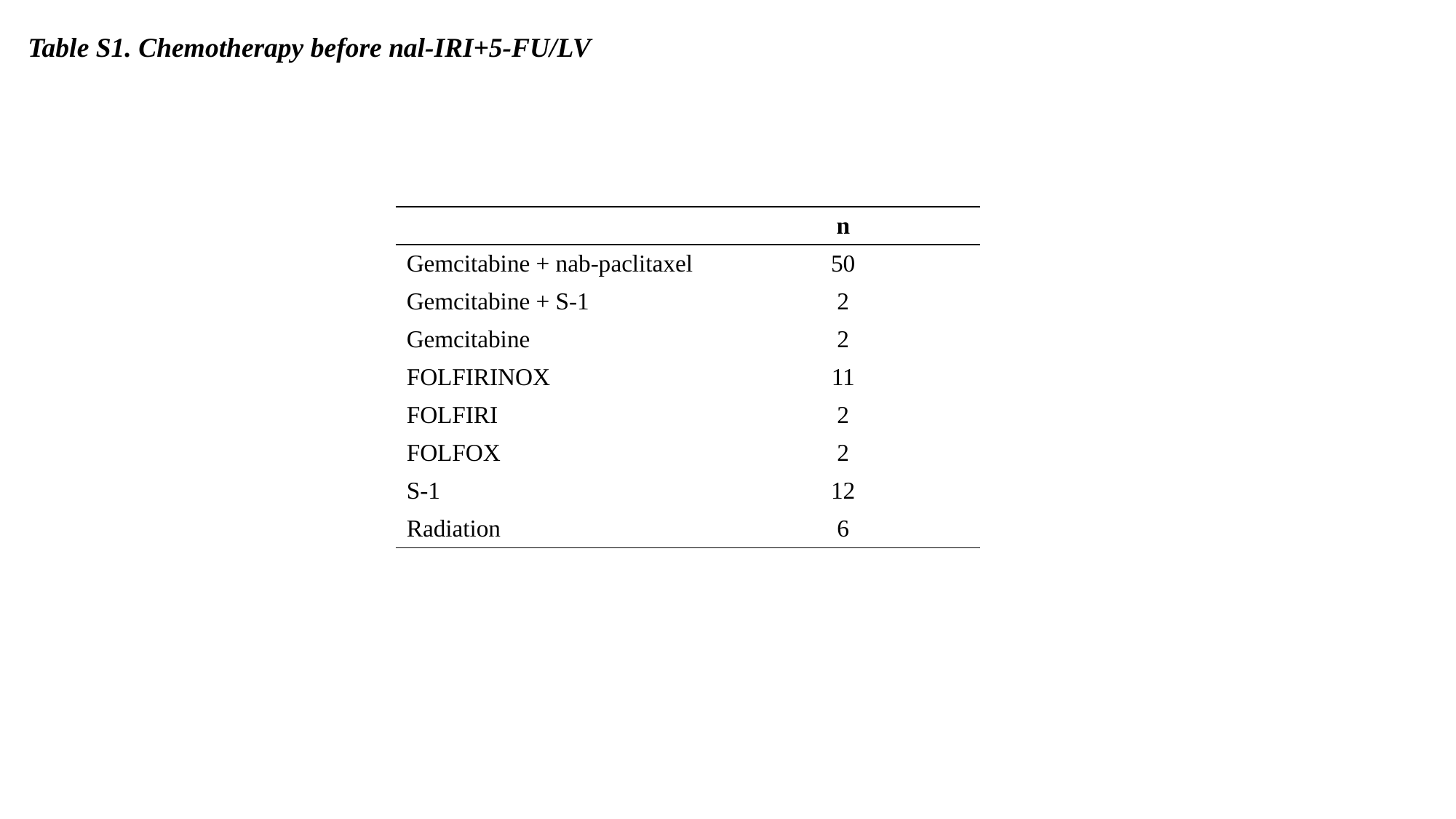

Table S1. Chemotherapy before nal-IRI+5-FU/LV
| | n |
| --- | --- |
| Gemcitabine + nab-paclitaxel | 50 |
| Gemcitabine + S-1 | 2 |
| Gemcitabine | 2 |
| FOLFIRINOX | 11 |
| FOLFIRI | 2 |
| FOLFOX | 2 |
| S-1 | 12 |
| Radiation | 6 |

## Slide 2
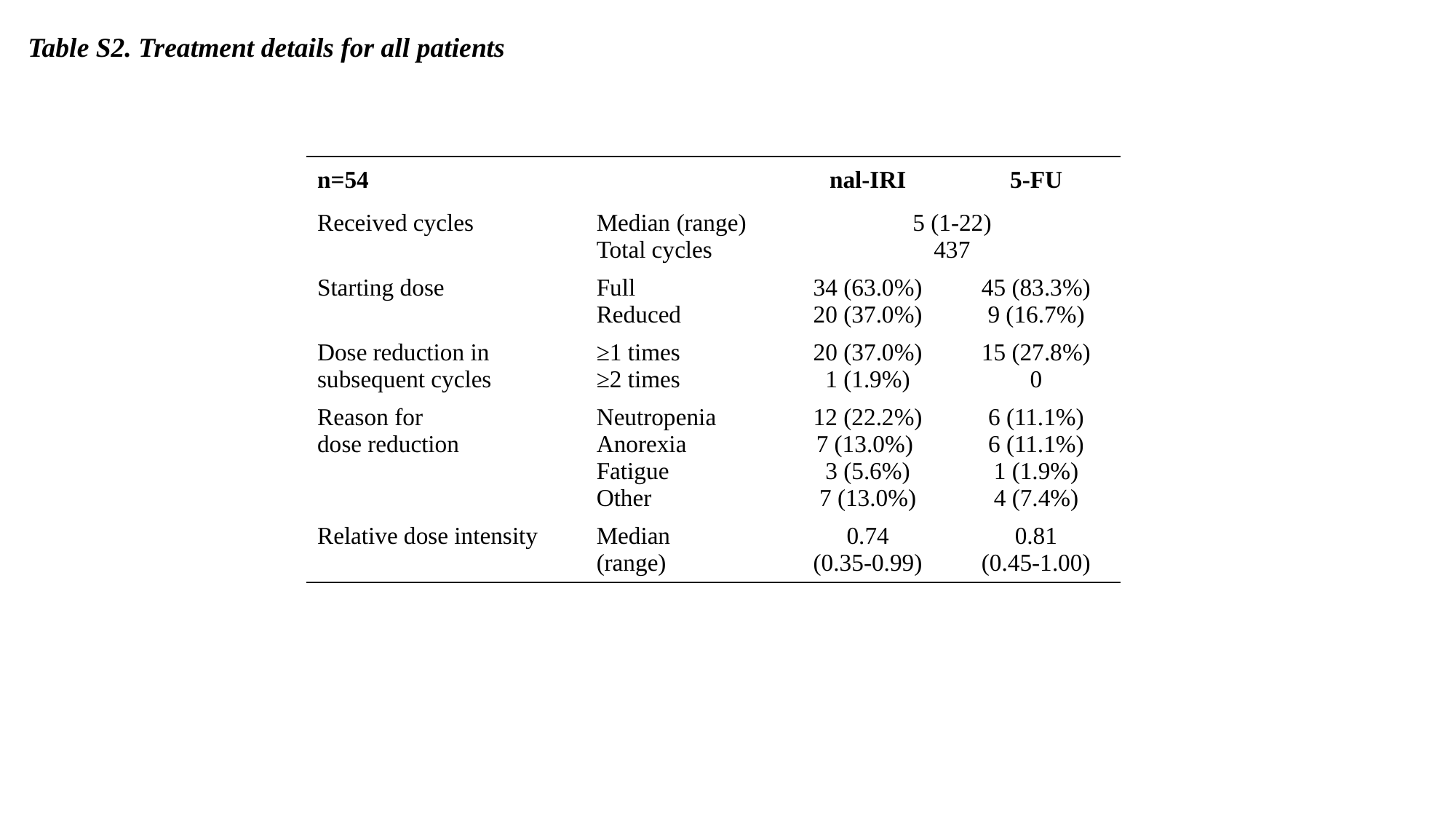

Table S2. Treatment details for all patients
| n=54 | | nal-IRI | 5-FU |
| --- | --- | --- | --- |
| Received cycles | Median (range) Total cycles | 5 (1-22) 437 | |
| Starting dose | Full Reduced | 34 (63.0%) 20 (37.0%) | 45 (83.3%) 9 (16.7%) |
| Dose reduction in subsequent cycles | ≥1 times ≥2 times | 20 (37.0%) 1 (1.9%) | 15 (27.8%) 0 |
| Reason for dose reduction | Neutropenia Anorexia Fatigue Other | 12 (22.2%) 7 (13.0%) 3 (5.6%) 7 (13.0%) | 6 (11.1%) 6 (11.1%) 1 (1.9%) 4 (7.4%) |
| Relative dose intensity | Median (range) | 0.74 (0.35-0.99) | 0.81 (0.45-1.00) |

## Slide 3
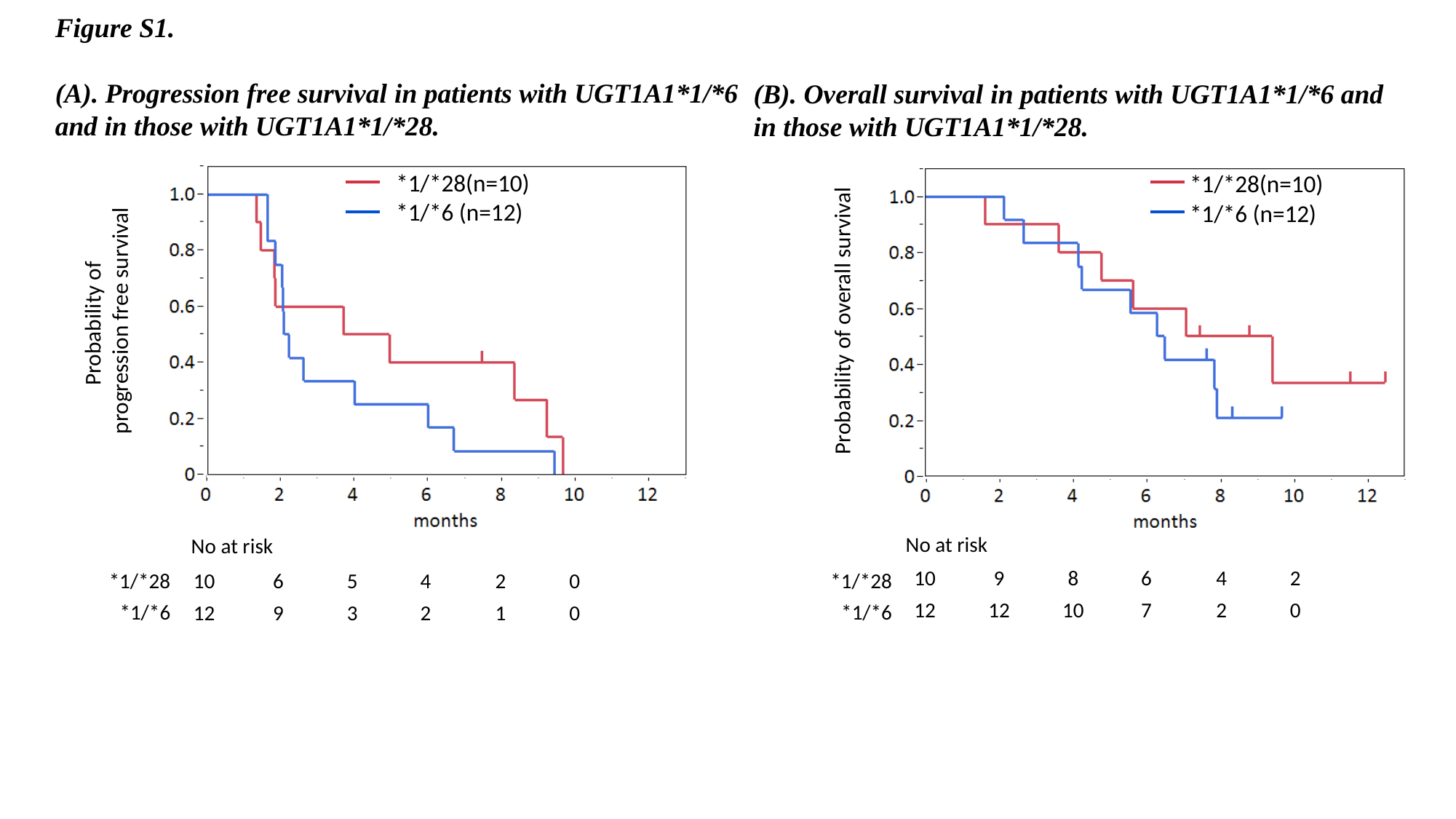

Figure S1.
(A). Progression free survival in patients with UGT1A1*1/*6 and in those with UGT1A1*1/*28.
(B). Overall survival in patients with UGT1A1*1/*6 and in those with UGT1A1*1/*28.
*1/*28(n=10)
*1/*6 (n=12)
*1/*28(n=10)
*1/*6 (n=12)
Probability of
progression free survival
Probability of overall survival
| No at risk |
| --- |
| No at risk |
| --- |
| 10 | | 9 | | 8 | | 6 | | 4 | | 2 | | |
| --- | --- | --- | --- | --- | --- | --- | --- | --- | --- | --- | --- | --- |
| 12 | | 12 | | 10 | | 7 | | 2 | | 0 | | |
| | | | | | | | | | | | | |
| 10 | | 6 | | 5 | | 4 | | 2 | | 0 | | |
| --- | --- | --- | --- | --- | --- | --- | --- | --- | --- | --- | --- | --- |
| 12 | | 9 | | 3 | | 2 | | 1 | | 0 | | |
| | | | | | | | | | | | | |
| \*1/\*28 |
| --- |
| \*1/\*6 |
| |
| |
| \*1/\*28 |
| --- |
| \*1/\*6 |
| |
| |

## Slide 4
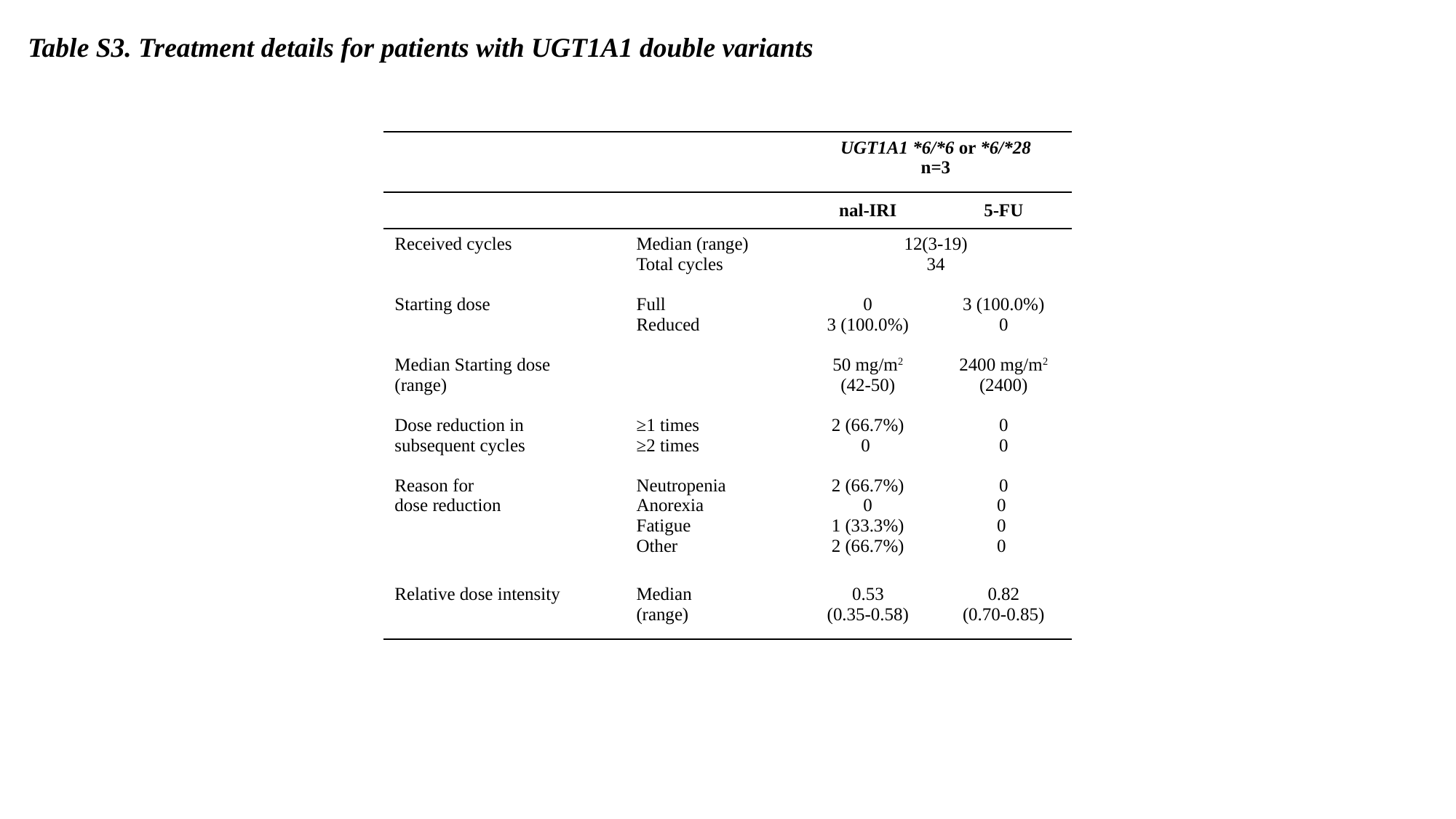

Table S3. Treatment details for patients with UGT1A1 double variants
| | | UGT1A1 \*6/\*6 or \*6/\*28 n=3 | |
| --- | --- | --- | --- |
| | | nal-IRI | 5-FU |
| Received cycles | Median (range) Total cycles | 12(3-19) 34 | |
| Starting dose | Full Reduced | 0 3 (100.0%) | 3 (100.0%) 0 |
| Median Starting dose (range) | | 50 mg/m2 (42-50) | 2400 mg/m2 (2400) |
| Dose reduction in subsequent cycles | ≥1 times ≥2 times | 2 (66.7%) 0 | 0 0 |
| Reason for dose reduction | Neutropenia Anorexia Fatigue Other | 2 (66.7%) 0 1 (33.3%) 2 (66.7%) | 0 0 0 0 |
| Relative dose intensity | Median (range) | 0.53 (0.35-0.58) | 0.82 (0.70-0.85) |

## Slide 5
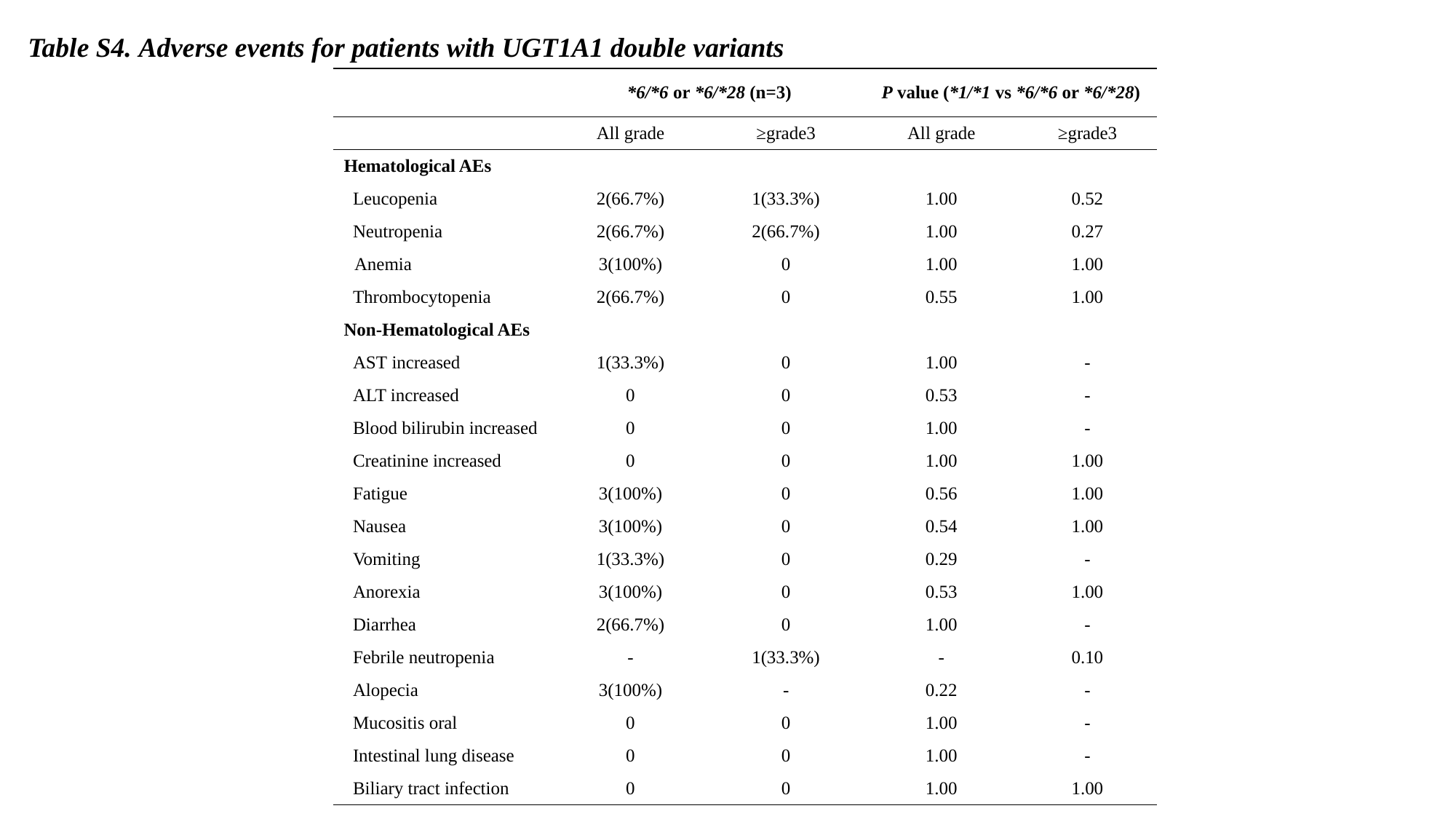

Table S4. Adverse events for patients with UGT1A1 double variants
| | \*6/\*6 or \*6/\*28 (n=3) | | P value (\*1/\*1 vs \*6/\*6 or \*6/\*28) | |
| --- | --- | --- | --- | --- |
| | All grade | ≥grade3 | All grade | ≥grade3 |
| Hematological AEs | | | | |
| Leucopenia | 2(66.7%) | 1(33.3%) | 1.00 | 0.52 |
| Neutropenia | 2(66.7%) | 2(66.7%) | 1.00 | 0.27 |
| Anemia | 3(100%) | 0 | 1.00 | 1.00 |
| Thrombocytopenia | 2(66.7%) | 0 | 0.55 | 1.00 |
| Non-Hematological AEs | | | | |
| AST increased | 1(33.3%) | 0 | 1.00 | - |
| ALT increased | 0 | 0 | 0.53 | - |
| Blood bilirubin increased | 0 | 0 | 1.00 | - |
| Creatinine increased | 0 | 0 | 1.00 | 1.00 |
| Fatigue | 3(100%) | 0 | 0.56 | 1.00 |
| Nausea | 3(100%) | 0 | 0.54 | 1.00 |
| Vomiting | 1(33.3%) | 0 | 0.29 | - |
| Anorexia | 3(100%) | 0 | 0.53 | 1.00 |
| Diarrhea | 2(66.7%) | 0 | 1.00 | ‐ |
| Febrile neutropenia | - | 1(33.3%) | - | 0.10 |
| Alopecia | 3(100%) | - | 0.22 | - |
| Mucositis oral | 0 | 0 | 1.00 | - |
| Intestinal lung disease | 0 | 0 | 1.00 | - |
| Biliary tract infection | 0 | 0 | 1.00 | 1.00 |

## Slide 6
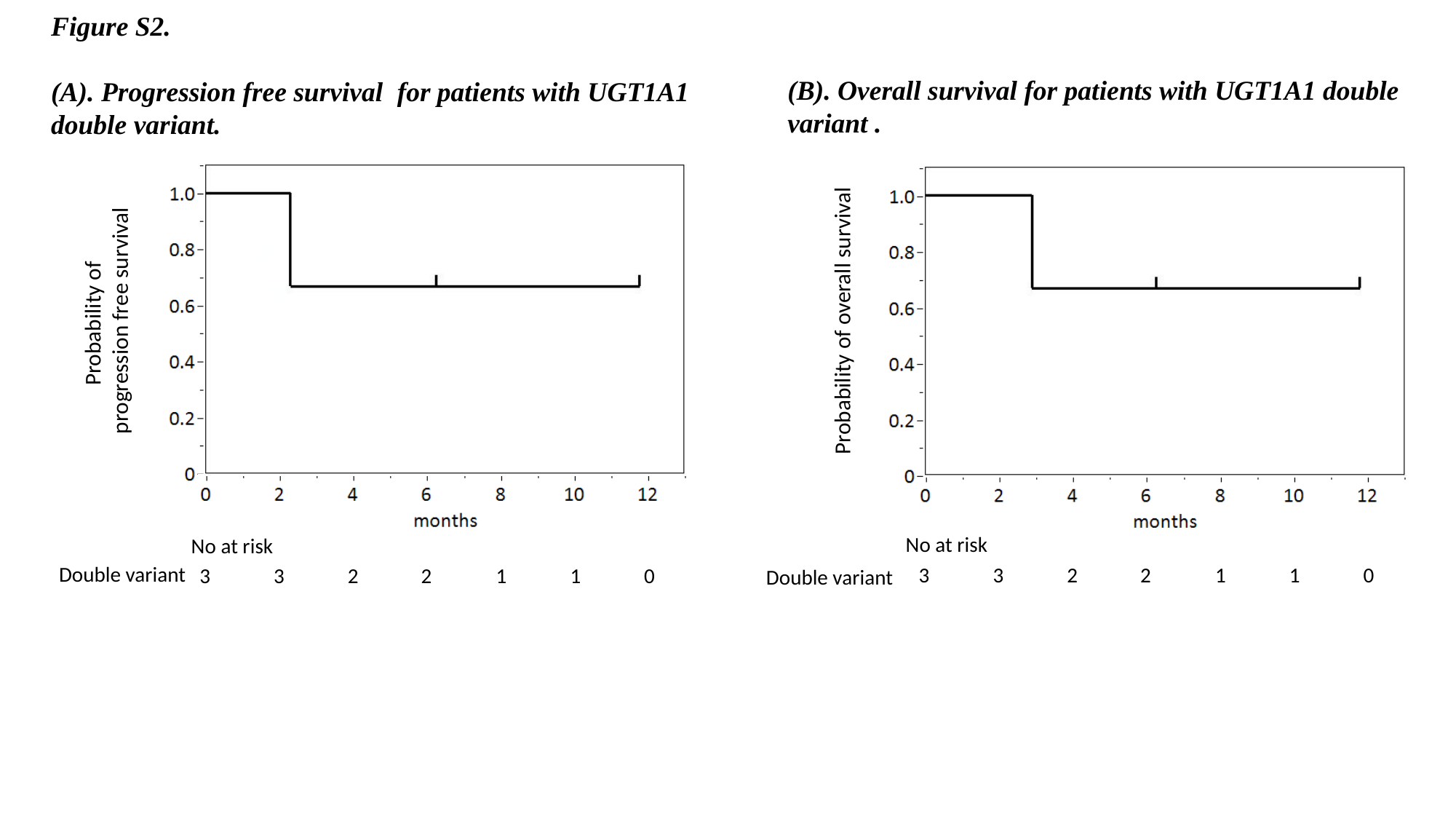

Figure S2.
(A). Progression free survival for patients with UGT1A1 double variant.
(B). Overall survival for patients with UGT1A1 double variant .
Probability of
progression free survival
Probability of overall survival
| No at risk |
| --- |
| No at risk |
| --- |
| Double variant |
| --- |
| |
| 3 | | 3 | | 2 | | 2 | | 1 | | 1 | | 0 |
| --- | --- | --- | --- | --- | --- | --- | --- | --- | --- | --- | --- | --- |
| 3 | | 3 | | 2 | | 2 | | 1 | | 1 | | 0 |
| --- | --- | --- | --- | --- | --- | --- | --- | --- | --- | --- | --- | --- |
| Double variant |
| --- |
| |
